# Triptolide has anticancer and chemosensitization effects by down-regulating Akt activation through the MDM2/REST pathway in human breast cancer

**DOI:** 10.18632/oncotarget.8207

**Published:** 2016-03-19

**Authors:** Jing Xiong, Tiefen Su, Zhiling Qu, Qin Yang, Yu Wang, Jiansha Li, Sheng Zhou

**Affiliations:** ^1^ Institute of Pathology, Tongji Hospital, Tongji Medical College, Huazhong University of Science and Technology, Wuhan 430030, China

**Keywords:** breast neoplasms, MDM2, Akt, triptolide, REST

## Abstract

Triptolide has been shown to exhibit anticancer activity. However, its mechanism of action is not clearly defined. Herein we report a novel signaling pathway, MDM2/Akt, is involved in the anticancer mechanism of triptolide. We observed that triptolide inhibits MDM2 expression in human breast cancer cells with either wild-type or mutant p53. This MDM2 inhibition resulted in decreased Akt activation. More specifically, triptolide interfered with the interaction between MDM2 and the transcription factor REST to increase expression of the regulatory subunit of PI3-kinase p85 and consequently inhibit Akt activation. We further showed that, regardless of p53 status, triptolide inhibited proliferation, induced apoptosis, and caused G1 phase cell cycle arrest. Triptolide also enhanced the cytotoxic effect of doxorubicin. MDM2 inhibition plays a causative role in these effects. The inhibitory effect of triptolide on MDM2-mediated Akt activation was eliminated with MDM2 overexpression. MDM2-overexpressing tumor cells, in turn, were less susceptible to the anticancer and chemosensitization effects of triptolide than control cells. Triptolide also exhibited anticancer and chemosensitization effects in nude mouse xenograft model. When it was administered to tumor-bearing nude mice, triptolide inhibited tumor growth and enhanced the antitumor effects of doxorubicin. In summary, triptolide has anticancer and chemosensitization effects by down-regulating Akt activation through the MDM2/REST pathway in human breast cancer. Our study helps to elucidate the p53-independent regulatory function of MDM2 in Akt signaling, offering a novel view of the mechanism by which triptolide functions as an anticancer agent.

## INTRODUCTION

The mouse double minute 2 (MDM2) protein is a multifunctional oncoprotein that has been suggested to play both p53-dependent and -independent roles in oncogenesis. MDM2 gained considerable attention following its identification as an important inhibitor of the tumor suppressor p53. The NH_2_ terminus of MDM2 protein binds to p53 and represses p53-mediated transcriptional activity [1], whereas its COOH terminus contains a RING finger domain that is an E3 ubiquitin ligase, thus promoting p53 ubiquitination and proteasomal degradation [2]. However, increasing evidence suggests that MDM2 also has activities independent of p53. In the presence or absence of functional p53, tumor cells which expressed high levels of MDM2 both show high invasive potential [3–5]. Although the interaction between MDM2 and p53 has been extensively investigated, the p53-independent oncogenic functions of MDM2 remains underexplored.

MDM2 interacts with several components of the PI3K/Akt/mTOR signaling pathway. Activation of PI3K upon growth factor stimulation leads to Akt-dependent phosphorylation of MDM2 on serine 166 and serine 186, which facilitates translocation of MDM2 from the cytoplasm into the nucleus. This is a crucial step in the mechanism through which MDM2 inhibits the transcriptional activity of p53 and targets p53 for degradation [6]. Moreover, phosphorylation of MDM2 by Akt protects MDM2 from self-ubiquitination and proteasomal degradation, thereby increasing MDM2 stability [7]. In addition, MDM2 expression may be enhanced by mTOR through the translational machinery [8]. The substrates of mTOR include the ribosomal protein S6 kinase (S6K) and the eukaryotic initiation factor 4E-binding protein 1 (4E-BP1), both of which are key regulators controlling the cap-dependent mRNA translation [9–11]. Interestingly, recent evidence suggests that MDM2 in turn up-regulates the PI3K/Akt/mTOR signaling pathway. MDM2 interferes with localization of the repressor element-1 silencing transcription factor (REST) on the promoter of the PI3K regulatory subunit p85, suppresses p85 expression, and enhances PI3K activity and thus Akt phosphorylation [12].

MDM2 gene amplification occurs in diverse human malignancies, including breast cancer [13–15]. A series of studies indicate that overexpression of MDM2 in tumors correlates with a poor prognosis for these cancer patients [16], and high MDM2 expression occurs more frequently in metastatic and recurrent cancers than in primary tumors [17]. Moreover, clinical observations establish the role of MDM2 in the development of chemoresistance [18–20]. Therefore, MDM2 has been considered to be a potential molecular target for cancer therapy.

As the central component of growth factor-mediated cell survival, PI3K/Akt/mTOR signaling is often constitutively activated in malignancy. Tumor growth and progression are associated with high levels of PI3K/Akt/mTOR activation. PI3K/Akt/mTOR activation has also been implicated in diminished sensitivity of cancer cells to chemotherapeutic drugs. Therefore, this signaling is a constitutive targeted pathway in human cancer therapy [21, 22].

For years, our research has gathered on the p53-independent activities and mechanisms of MDM2 in oncogenesis [4, 5]. We also try to screen for potential small molecule inhibitors that specifically target MDM2 for human cancer therapy [5, 23], and find several promising natural products, including triptolide. Triptolide is a natural product extracted from the native Chinese herb *Tripterygium wilfordii* Hook.f. *Tripterygium wilfordii* Hook.f has been used for centuries to treat autoimmune diseases [24]. Triptolide is recently reported to exhibit potent anticancer activity by suppressing proliferation and inducing apoptosis in a broad range of human cancers [25, 26]. Various proliferation or antiapoptotic factors have been implicated in the biological effects of triptolide, however, its primary molecular target and mechanism of action remain to be clarified. We observed that triptolide inhibits MDM2 expression in tumor cells with either wild-type or mutant p53. This MDM2 inhibition by triptolide results in decreased Akt activation, which made us further interested in the possible relationship between MDM2 and Akt implicated in the biological effects of triptolide. In the present study, we have shown that triptolide interferes with the interaction between MDM2 and the transcription factor REST to increase expression of the regulatory subunit of PI3-kinase p85 and consequently inhibit Akt activation. Further, triptolide has anticancer and chemosensitization effects *in vitro* and *in vivo*. Inhibition of MDM2 plays a causative role in these effects of triptolide. Our study helps to elucidate the p53-independent regulatory function of MDM2 in Akt signaling and a novel mechanism by which triptolide functions as an anticancer agent.

## RESULTS

### Triptolide inhibits MDM2 expression in human breast cancer cell lines

**Figure 1:**
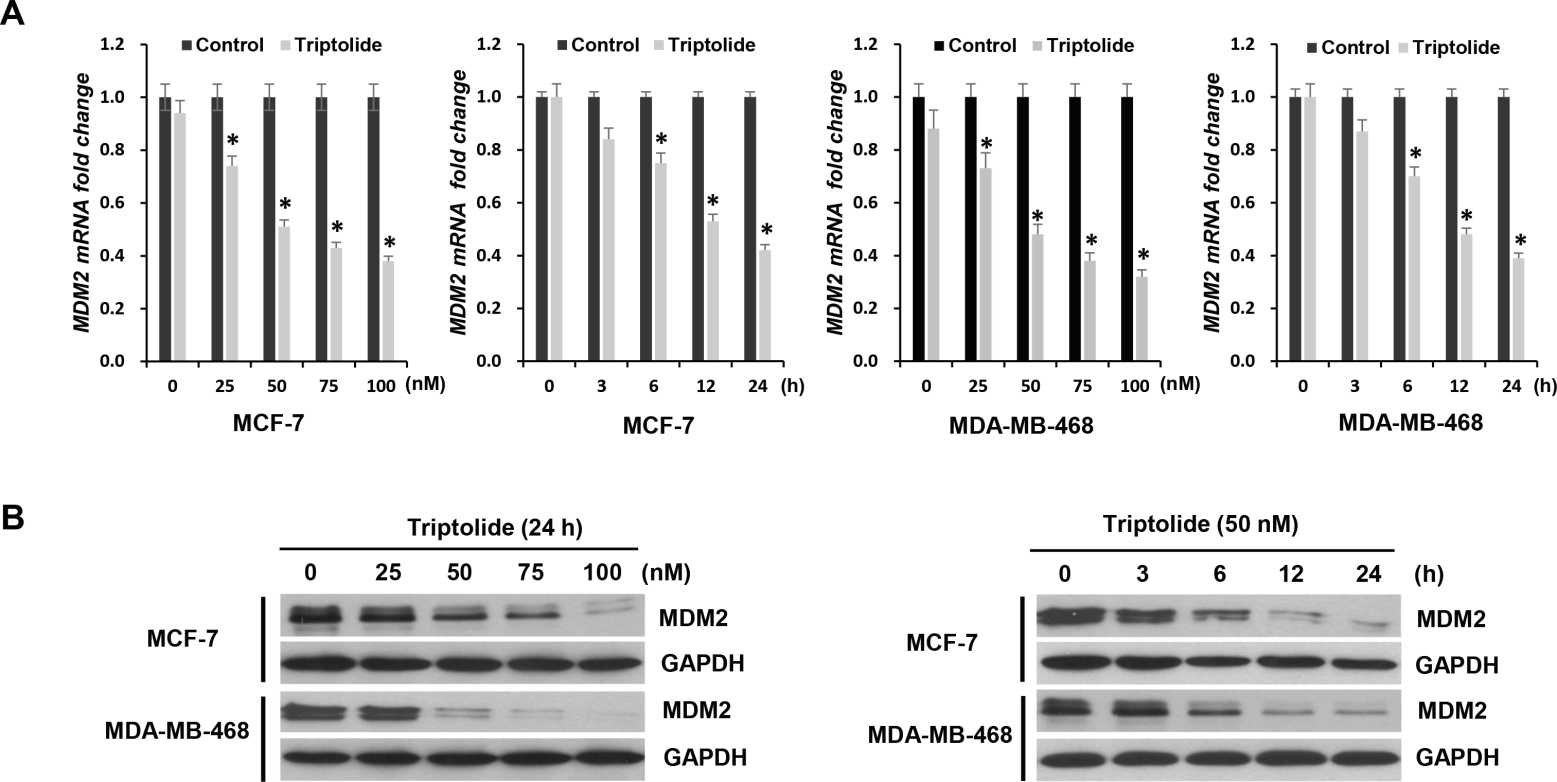
The inhibitory effect of triptolide on MDM2 expression (**A**) Quantitative RT-PCR for MDM2 mRNA expression in MCF-7 and MDA-MB-468 cells treated with either different concentrations of triptolide for 24 hours or 50 nM triptolide for different times, as indicated. Data represent means ± SEM of three independent experiments normalized to GAPDH. In both MCF-7 and MDA-MB-468 cells, triptolide significantly inhibited MDM2 mRNA expression. The inhibitory effect of triptolide on MDM2 mRNA expression was shown in a dose- and time-dependent manner. *P < 0.05. (**B**) Western blot assays showing the dose-response and time-course of MDM2 inhibition by triptolide. GAPDH served as an internal control for equal protein loading.

We first investigated the effect of triptolide on MDM2 expression. To determine if the effect of triptolide is dependent on p53, we compared the results in the paired MCF-7 (with wild-type p53) and MDA-MB-468 (p53 mutant) cell lines. After incubation with triptolide at various concentrations, cell lysates were collected at different times and measured by quantitative RT-PCR analysis for MDM2 expression. As shown in Figure 1A, in MCF-7 cells that contain wild-type p53, triptolide significantly inhibited MDM2 mRNA expression. Further, the inhibitory effect of triptolide on MDM2 mRNA expression was shown in a dose- and time-dependent manner. Also in MDA-MB-468 cells that contain mutant p53, the mRNA levels of MDM2 were significantly inhibited by triptolide. And the inhibitory effect was also shown in a dose- and time-dependent manner.

Next, we examined the effect of triptolide on MDM2 expression at protein level in these two human breast cancer cell lines. Similarly, regardless of p53 status, triptolide inhibited MDM2 protein expression, and likewise, the inhibitory effect of triptolide on MDM2 protein expression was shown in a dose- and time-dependent manner (Figure 1B). These results were consistent with the changes at mRNA level. Therefore, triptolide inhibits MDM2 expression at mRNA as well as protein levels, independent of the p53 status of the tumor cells.

### Triptolide inhibits MDM2-mediated Akt activation

**Figure 2:**
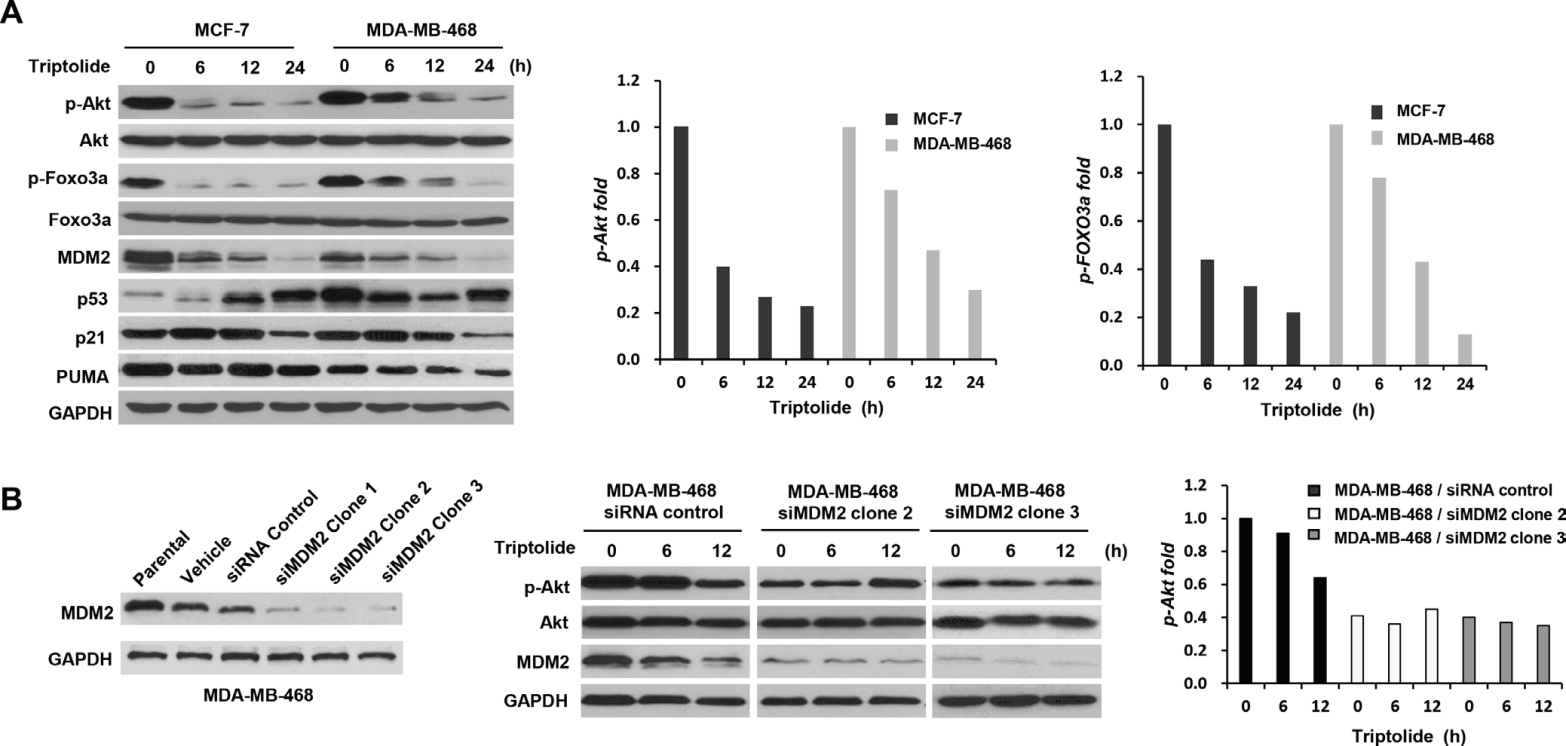
The inhibitory effect of triptolide on MDM2-mediated Akt activation (**A**) Western blot assays showing the inhibitory effect of triptolide on Akt activity. MCF-7 and MDA-MB-468 cells were treated with 50 nM triptolide for different times. Cell extracts were tested by Western blot assay for the expression of MDM2, p53, p21, PUMA, Akt, phosphorylated Akt (S473), Foxo3a and phosphorylated Foxo3a. Phosphorylated Akt and phosphorylated Foxo3a were dramatically decreased following treatment with triptolide. The quantification results are shown on the graphs to the right. (**B**) MDM2 plays a role in the reduction of Akt activation in triptolide-treated tumor cells. MDA-MB-468 cells were transfected with pSUPER/MDM2 siRNA (clone1, 2, and 3) or pSUPER/control siRNA plasmids. Expressions of MDM2 in parent cells and cells transfected with the pSUPER vector only (vehicle), pSUPER containing a control siRNA (siRNA control), or pSUPER/MDM2 siRNA (clone1, 2, and 3) were detected by Western blotting. The kinetic expression of phosphorylated Akt/total Akt protein following triptolide treatment was then studied in MDA-MB-468 cells transfected with either control siRNA or MDM2 siRNA (clone2 and 3). Knocking down MDM2 expression by siRNA greatly impaired the inhibitory effect of triptolide on Akt activity.

Inhibition of MDM2 by triptolide led to increased p53 accumulation; however, p53 function was not activated. Triptolide failed to increase and even decreased the expression of p53 target protein p21 and PUMA (Figure 2A). We further observed that triptolide inhibited Akt activation, as manifested by the changes in the phosphorylation status of Akt. Although the total protein level remained unchanged, phosphorylated Akt was dramatically decreased following treatment with triptolide (Figure 2A). And this defect in Akt phosphorylation/activation was companied by impaired Akt activity toward its substrate Foxo3a. Phosphorylation of Foxo3a was also decreased in tumor cells treated with triptolide (Figure 2A). More importantly, consistent with MDM2 inhibition, the inhibitory effect on Akt activity by triptolide was observed in both MCF-7 and MDA-MB-468 cells, suggesting a p53-independent mechanism.

To determine whether MDM2 plays a role in the reduction of Akt activation in triptolide-treated tumor cells, MDA-MB-468 cells were transfected with MDM2 siRNA, followed by exposure to triptolide. As shown in Figure 2B, knocking down MDM2 expression by siRNA greatly impaired the inhibitory effect of triptolide on Akt activity. Therefore, MDM2 was required for the down-regulation of Akt activity by triptolide. Additionally, it is worthwhile to note that MDM2 siRNA resulted in decreased cellular level of phosphorylated Akt (Figure 2B).

### Triptolide interferes with the interaction between MDM2 and REST to increase the expression of p85, the regulatory subunit of PI3-kinase

**Figure 3:**
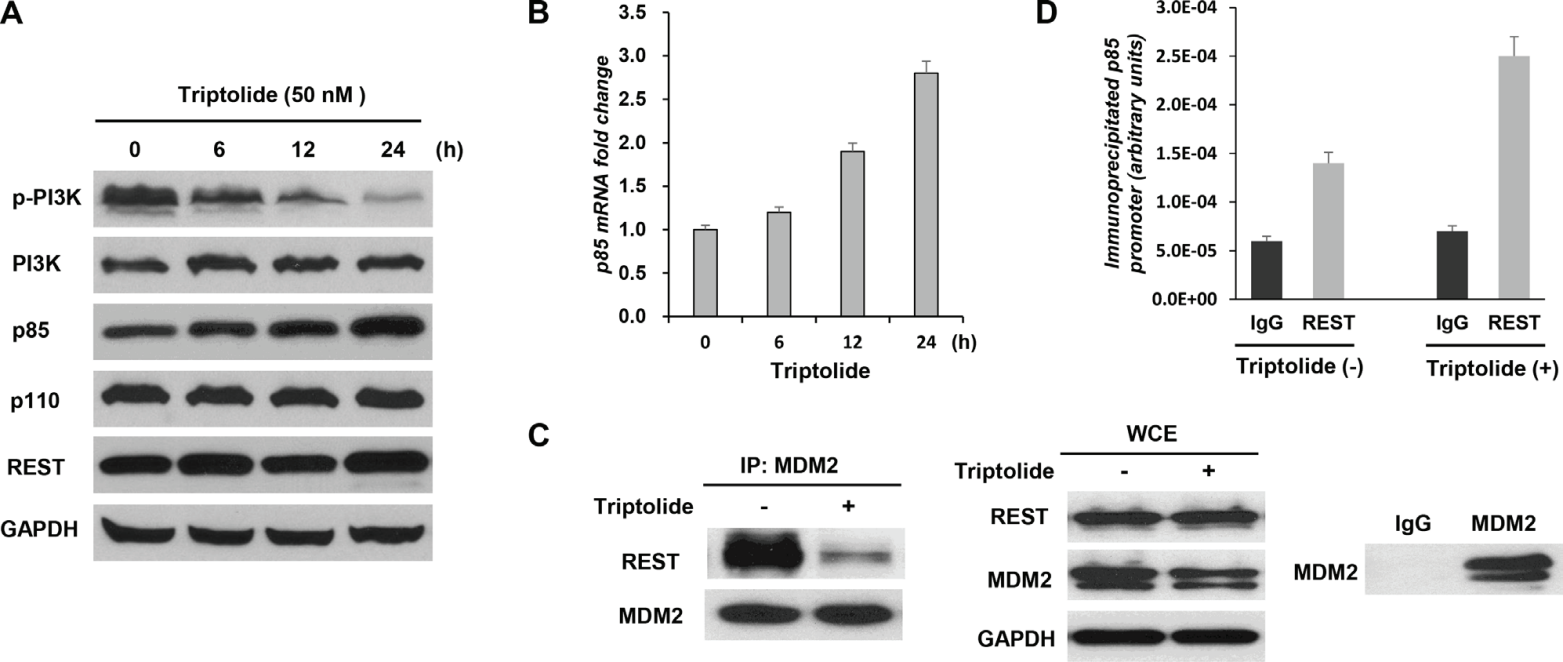
Triptolide interferes with the interaction between MDM2 and REST to increase expression of the regulatory subunit of PI3-kinase p85 (**A** and **B**) Triptolide up-regulates p85 expression. MDA-MB-468 cells were treated with 50 nM triptolide for different times. Cell extracts were tested by Western blot assay for the expression of PI3K, phosphorylated PI3K, p85, p110 and REST (A). Expression of p85 transcript was determined by quantitative RT-PCR (B). The levels of both p85 protein and transcript were significantly increased following treatment with triptolide. (**C**) Triptolide suppresses physical interaction between MDM2 and REST proteins. MDA-MB-468 cells were treated with 50 nM triptolide for 3 hours. MDM2 was immunoprecipitated, and REST co-purification was analyzed by Western blotting. The counterblot for MDM2 shows equal immunoprecipitation of MDM2. WCEs show cellular levels of proteins after triptolide treatment. Immunoprecipitations using normal mouse IgG antibody are included as controls. (**D**) Triptolide enhances localization of REST on p85 promoter. Triptolide-treated MDA-MB-468 cells were subjected to chromatin immunoprecipitation (CHIP) analysis of p85 promoter using a REST antibody.

Akt activation is a downstream event of phosphorylation of phosphatidylinositol-4, 5-bisphosphate (PIP2) to phosphatidylinositol-3, 4, 5-phosphate (PIP3) by PI3-kinase [27]. We therefore investigated the effect of triptolide on PI3-kinase. Results showed that triptolide also inhibited PI3-kiase activity. Phosphorylated PI3-kinase was significantly decreased following treatment with triptolide (Figure 3A).

PI3-kinae is a heterodimeric signaling factor composed of a p110 catalytic subunit and a p85 regulatory subunit [28]. As the negative regulator of PI3-kinase, p85 interacts with p110 and suppresses its catalytic activity [29]. We further determined the expression of p85 and p110. No obvious change in p110 expression was observed (Figure 3A), whereas the levels of both p85 protein (Figure 3A) and transcript (Figure 3B) were significantly increased after triptolide treatment.

Repressor element-1 silencing transcription factor (REST), a transcriptional regulator, has been reported to exhibit a tumor suppressor function in diverse human malignancies, including human breast cancer [30–32]. P85 has been identified as a direct target of REST. Research shows that MDM2 may interfere with the tumor suppressor function of the transcription factor REST to inhibit p85 expression and thus to activate Akt phosphorylation [12]. To further decipher the mechanism of MDM2-mediated Akt inactivation by triptolide, the possible involvement of REST was studied. Significantly, triptolide did not alter REST expression (Figure 3A), but influenced REST function. Immunoprecipitation (IP) assay results demonstrated that triptolide treatment suppressed physical interaction between MDM2 and REST proteins (Figure 3C). MDA-MB-468 cells were treated with 50 nM triptolide for 3 hours. This condition allowed us to investigate the interaction between MDM2 and REST proteins without influence of decreased MDM2 level following treatment with triptolide. MDM2 was immunoprecipitated, and REST co-purification was analyzed by Western blotting. A significant decrease in MDM2-REST co-purification was observed in response to triptolide treatment. The Whole cell extracts (WCEs) showed no difference in protein expression levels after 3 hours of triptolide. And control immunoprecipitations using normal mouse IgG show specificity in these experiments. On the other hand, chromatin immunoprecipitation (CHIP) assay results demonstrated that triptolide treatment enhanced localization of REST on the p85 promoter (Figure 3D). Taken together, these results indicate that triptolide interferes with the interaction between MDM2 and REST to increase expression of the regulatory subunit of PI3-kinase p85 and consequently inhibit Akt activation.

### Triptolide has anticancer effects on cell proliferation, cell apoptosis, and cell cycle distribution, and the *in vitro* anticancer activities of triptolide are associated with its capacity to inhibit MDM2

**Figure 4:**
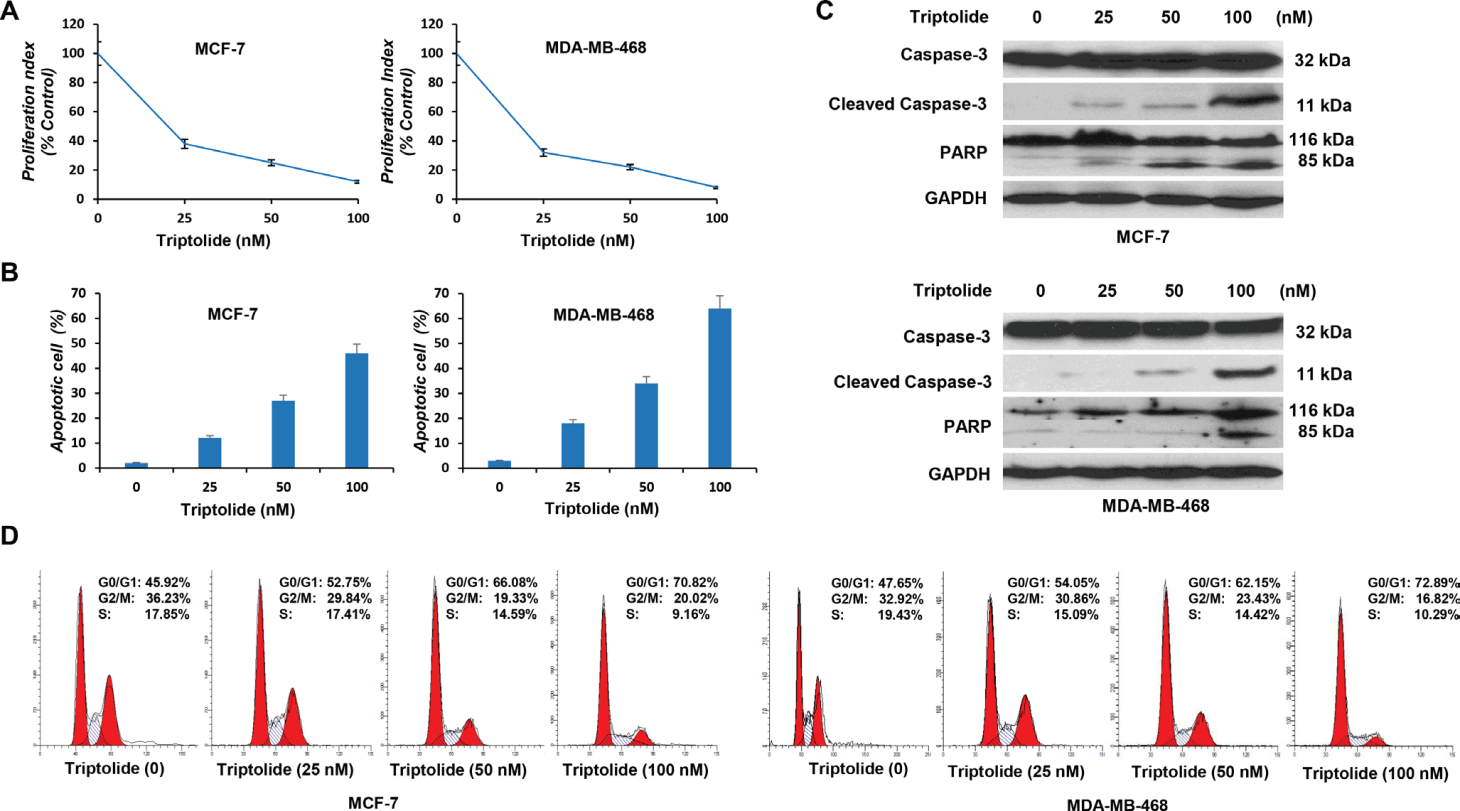
*In vitro* anticancer activities of triptolide MCF-7 and MDA-MB-468 cells were incubated with different concentrations of triptolide for 24 hours followed by cell proliferation assay (**A**), cell apoptosis assay (**B**), Western blot (**C**) and cell cycle distribution analysis (**D**). Data represent means ± SEM of three independent experiments.

Next, we investigated the biological significance of triptolide-induced MDM2 inhibition. In initial experiments, we examined the effect of triptolide on the proliferation of tumor cells. Tumor cells were exposed to increasing concentrations of triptolide for 24 hours and cell viability was measured by WST-1 assay. Triptolide inhibited cell proliferation in both wild-type p53-expressing MCF-7 cells and mutated p53-expressing MDA-MB-468 cells, and the inhibition effect showed as a dose-dependent manner. As the concentration of triptolide increased, the number of viable cells gradually decreased (Figure 4A).

We then examined its effect on cell apoptosis. Apoptotic cells were stained with Annexin V and were quantitated by flow cytometry. Triptolide induced cell apoptosis in both MCF-7 and MDA-MB-468 cells. The percentage of apoptotic cells gradually increased, as the concentration of triptolide increased (Figure 4B). Correspondingly, expressions of cleaved caspase-3 and PARP were detected by Western blotting (Figure 4C).

In addition, we conducted cell cycle analysis. Cell cycle distribution was evaluated by flow cytometric determination of cellular DNA content. Triptolide induced G_1_ phase cell cycle arrest in both MCF-7 and MDA-MB-468 cells. The proportion of G_0_/G_1_ phase cells increased following treatment with triptolide, while cells in S phase and G_2_/M phase decreased accordingly (Figure 4D). These results together show that, regardless of the p53 status, triptolide inhibits cell proliferation, induces cell apoptosis, and causes G_1_ phase cell cycle arrest.

**Figure 5:**
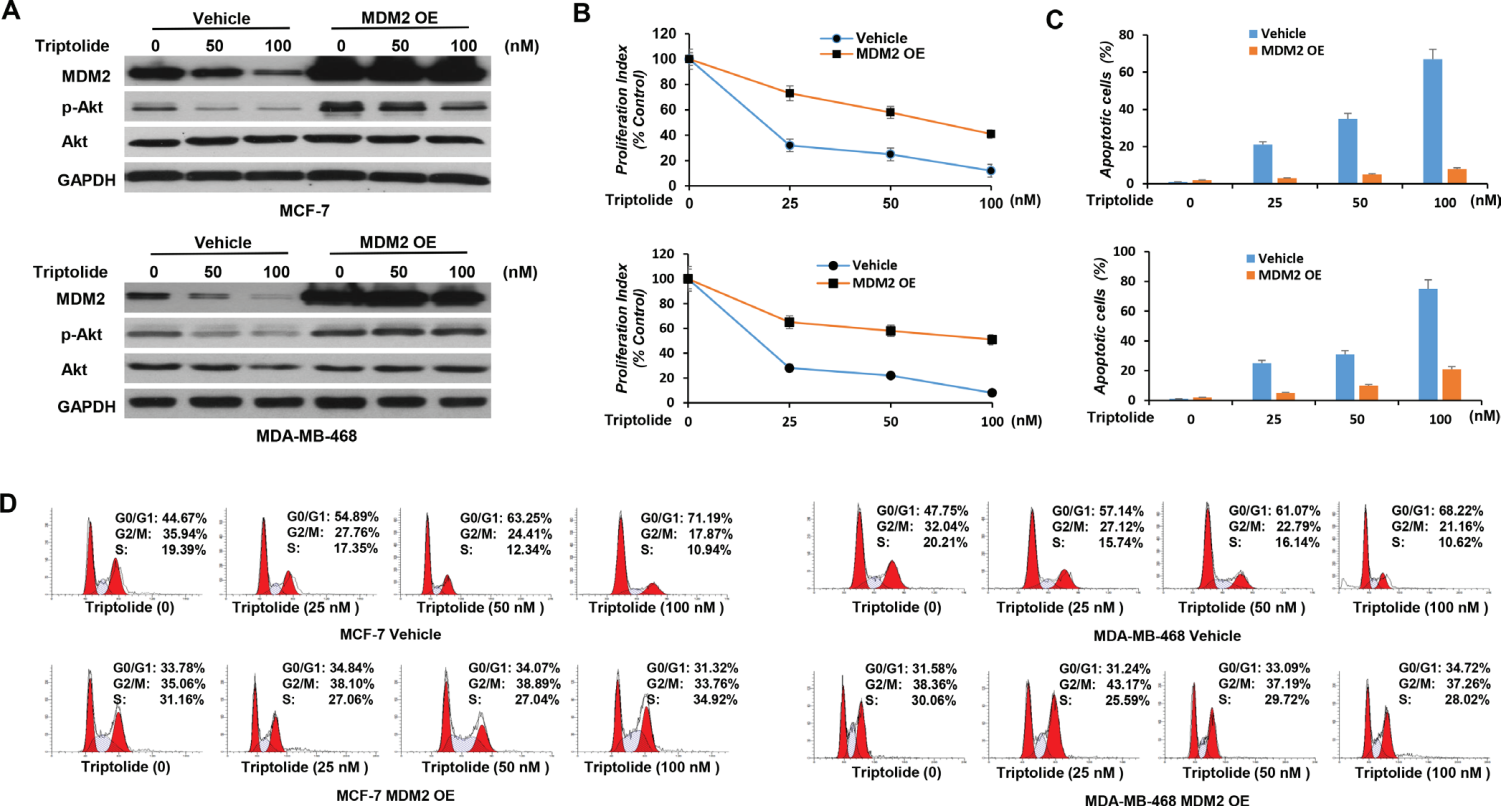
*In vitro* anticancer activities of triptolide are associated with its capacity to inhibit MDM2 (**A**) MCF-7 and MDA-MB-468 cells with stable overexpression of MDM2 were incubated with different concentrations of triptolide for 24 hours followed by Western blot analyses for the expression of MDM2, Akt and phosphorylated Akt (S473). GAPDH served as an internal control for equal protein loading. (**B**, **C** and **D**) MCF-7 and MDA-MB-468 cells with MDM2 overexpression were incubated with different concentrations of triptolide for 24 hours followed by cell proliferation assay (B), cell apoptosis assay (C), and cell cycle distribution analysis (D). Data represent means ± SEM of three independent experiments. MDM2-overexpressing tumor cells were less sensitive to the anticancer activities of triptolide than control cells.

However, MDM2 overexpression reduced these activities. As shown in Figure 5, MDM2-expressing plasmid was introduced into tumor cells. The inhibitory effect of triptolide on MDM2-mediated Akt activation was eliminated in tumor cells with MDM2 overexpression (Figure 5A). MDM2-overexpressing tumor cells, in turn, were less susceptible to the anticancer activities of triptolide than control cells (Figure 5B, 5C and 5D).

### Triptolide sensitizes tumor cells to chemotherapy

**Figure 6:**
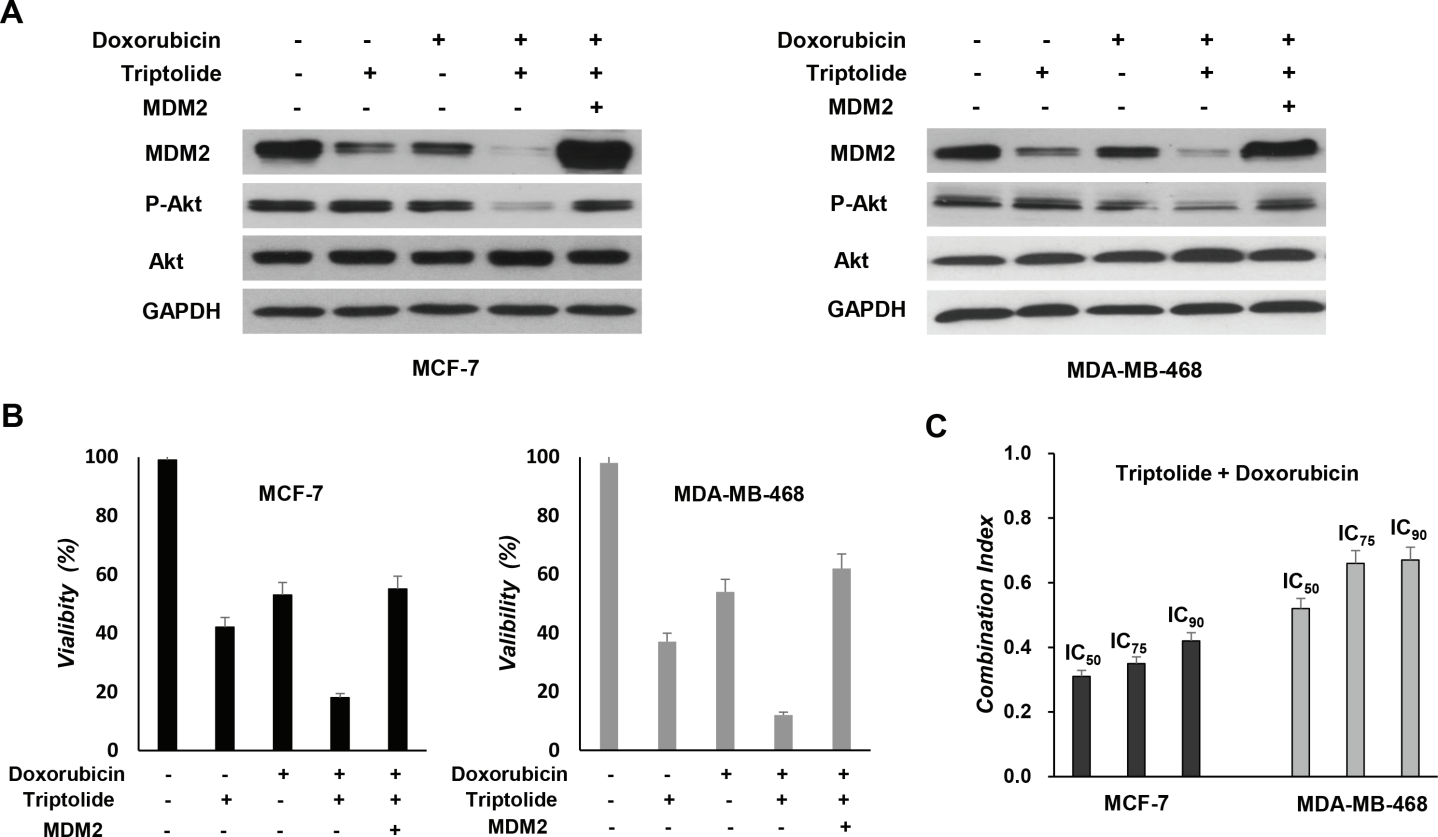
*In vitro* chemosensitization effect of triptolide (**A**) MCF-7 and MDA-MB-468 cells with MDM2 overexpression were pretreated with triptolide for 24 hours and then treated with doxorubicin (5 μM) for an additional 48 hours followed by Western blot analyses for the expression of MDM2, Akt and phosphorylated Akt (S473). (**B**) MCF-7 and MDA-MB-468 cells with MDM2 overexpression were pretreated with triptolide for 24 hours and then treated with doxorubicin (5 μM) for an additional 48 hours followed by WST-1 assay for cell viability. Triptolide treatment sensitizes tumor cells to doxorubicin-induced cell death. However, MDM2-overexpressing tumor cells were less sensitive to triptolide plus doxorubicin combination treatment. (**C**) Doxorubicin was combined with triptolide at a fixed ratio that spanned the individual IC_50_ of each drug. Data were analyzed by the method of Chou and Talalay to determine the combination index (CI) values. Results shown represent mean ± SEM from three independent experiments.

Doxorubicin is one of the most active chemotherapeutic agents for the therapy of human breast cancer. To evaluate the possible chemosensitization effect of triptolide, tumor cells were treated with triptolide and doxorubicin, either individually or in combination. WST-1 assay showed a synergistic interaction between triptolide and doxorubicin in induction of cell death. When combined with triptolide, the effect of doxorubicin was greatly enhanced (Figure 6A and 6B). To obtain more objective and complete data on the synergistic effect, combination index (CI) analysis was further performed. As shown in Figure 6C, the CI values at IC_50_, IC_75_ and IC_90_ levels were consistently less than 1.0, indicating a synergistic interaction.

We also investigated whether MDM2 inhibition plays a causative role in the chemosensitization effect of triptolide. Tumor cells were treated with triptolide and doxorubicin, either in the presence or absence of ectopic expression of MDM2. Tumor cells with MDM2 overexpression were less susceptible to triptolide plus doxorubicin combination treatment (Figure 6A and 6B). Thus, consistent with its *in vitro* anticancer activities, the chemosensitization effect of triptolide is also associated with its capacity to inhibit MDM2.

### *In vivo* MDM2 inhibition by triptolide shows anticancer activity and chemosensitization

**Figure 7:**
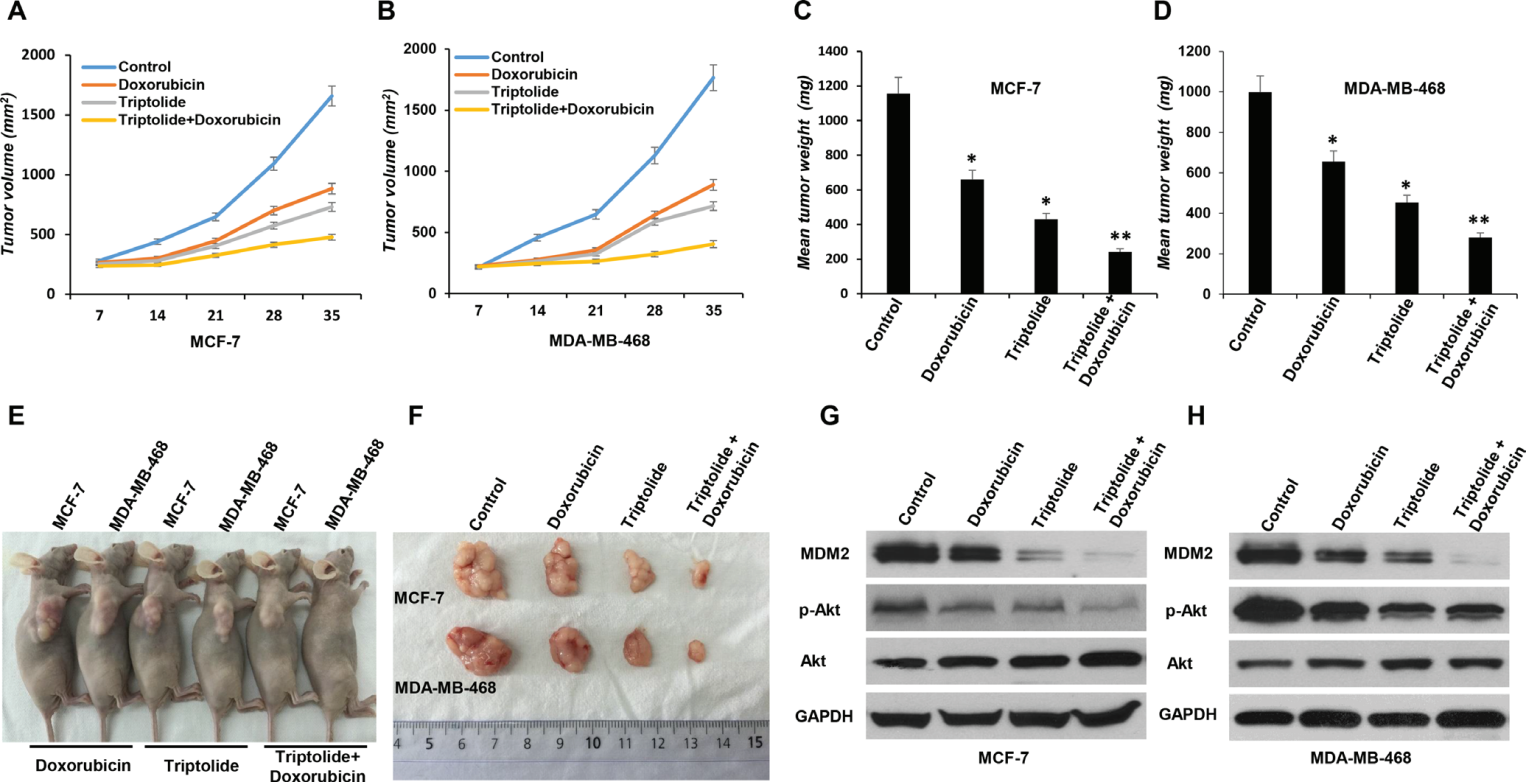
*In vivo* anticancer activity of triptolide administrated alone or in combination with doxorubicin in breast cancer xenograft models MCF-7 or MDA-MB-468 cells were subcutaneously implanted into nude mice and were subsequently treated with triptolide alone or in combination with doxorubicin. All mice underwent monitoring of tumor growth. The tumor size was measured twice weekly for 6 weeks. The mice were sacrificed on day 42, and the tumors were resected, weighted, and prepared for MDM2 expression and Akt activity analysis. (**A** and **B**) Xenograft tumor size. (**C** and **D**) Xenograft tumor weight. (**E** and **F**) Representative pictures from each treatment group. (**G** and **H**) MDM2 expression and Akt activity (phosphorylated Akt/total Akt) in tumor tissue studied by Western blotting. GAPDH served as an internal control for equal protein loading. Data represent means ± SEM; *n* = 10 mice/group. *P < 0.05.

Finally, to evaluate the biological relevance of our *in vitro* observation, we investigated whether *in vivo* MDM2 inhibition by triptolide also exhibits anticancer activity and chemosensitization effect in nude mouse xenograft model. Breast cancer xenograft model was established and was given triptolide treatment. Tumor growth indices, including tumor size, tumor weight, and inhibition rate of tumor growth, were continuously monitored and comparatively analyzed. In both MCF-7 and MDA-MB-468 xenograft models, triptolide showed strongly inhibitory effect on tumor growth. Compared to control tumors, triptolide suppressed xenograft tumor growth by 37.1% in MCF-7 xenograft model and 45.4% in MDA-MB-468 xenograft model, respectively (Figure 7A, 7B, 7C, 7D, 7E and 7F).

We also compared the response of xenograft tumor to single agent or combination chemotherapy. We found that, when combined with triptolide, the inhibitory effect of doxorubicin on tumor growth was greatly enhanced. In both MCF-7 and MDA-MB-468 xenograft models, triptolide plus doxorubicin combination treatment more effectively inhibited xenograft tumor growth than doxorubicin alone (MCF-7 xenograft model: inhibition rate of tumor growth 57.1% and 28.1%, respectively; MDA-MB-468 xenograft model: inhibition rate of tumor growth 65.6% and 20.8%, respectively) (Figure 7A, 7B, 7C, 7D, 7E and 7F).

Analysis of tumors collected at the end of the experiment confirmed the inhibitory effect of triptolide on MDM2 production and consequent Akt activation *in vivo*. MDM2 expression and Akt activity (phosphorylated Akt/total Akt) in tumor tissue were studied by Western blot assay. In accordance with *in vitro* results, regardless of p53 status, triptolide significantly inhibited MDM2 expression and Akt phosphorylation/activation *in vivo* (Figure 7G and 7H).

## DISCUSSION

There are multiple mechanisms for the biological activities of triptolide. However, details about these mechanisms and the primary molecular targets remain to be clarified. Our observations suggest a novel signaling pathway, MDM2/Akt, is involved in the anticancer mechanism of triptolide. We found that, independent of p53, triptolide significantly inhibited MDM2 expression, which resulted in decreased Akt activation, potent anticancer activities and chemosensitization.

Here, it was the p53-independent property of triptolide that attracted our attention. Inhibition of MDM2 by triptolide was observed in tumor cells with either wild-type or mutant p53. Although inhibition of MDM2 by triptolide led to increased p53 accumulation, the potent anticancer activities induced by triptolide are mostly unassociated with the function of p53. It is has been established that the major anticancer function of p53 is to arrest cell cycle in G1 phase and induce cell apoptosis through induction of its transcriptional targets p21 and PUMA. We tested for the expression of p21 and PUMA, and found that triptolide failed to increase and even decreased the expression of p21 and PUMA. Accumulation of p53 was likely due to release of MDM2-mediated degradation [33].

Although MDM2's main partner is p53, it also has p53-independent functions. For example, evidence suggests that MDM2 can also contribute to G1/S transition of the cell cycle by a mechanism independent of p53-interaction and regulation of endogenous p21 [34, 35]. We believe that the potent anticancer activities induced by triptolide are predominantly attributable to inhibition of MDM2-mediated Akt activation. Akt is major effector kinase relaying signaling events downstream of the PI3K pathway. It serves as a central node for the regulation of cell survival, proliferation and differentiation. Akt has a positive role for S phase entry, and deregulated Akt activation inhibits apoptosis and promotes cancerous growth and invasion [36]. In this study, we demonstrated that triptolide inhibited MDM2-mediated Akt activation. Triptolide treatment inhibited Akt phosphorylation/activation, which was companied by impaired Akt activity toward its substrate Foxo3a. Further, results showed that triptolide interfered with the interaction between MDM2 and the transcription factor REST to increase expression of the regulatory subunit of PI3-kinase p85 and consequently inhibit Akt activation, representing a novel mechanism of its action. More notable, the decrease in MDM2-REST interaction is an event that occurs early in response to triptolide treatment. Our results provide further evidence of the p53-independent regulatory function of MDM2 in Akt signaling. More than 50% of all solid tumors carry p53 mutation [37]. And there is a higher incidence of p53 mutation or loss of p53 expression observed in aggressive cancer [38]. Studies have indicated that alterations of the p53 gene are associated with the progression of human malignancy [39]. Therefore, it is significant to study the p53-independent role of MDM2. As Akt phosphorylates MDM2, MDM2-mediated Akt phosphorylation/activation may constitute a positive feedback loop responsible for constitutively activated Akt signaling. MDM2 therefore appears to be a pivotal target in cancer therapy, particularly for the high-risk, refractory cancers.

MDM2 has been considered as a target for human cancer therapy. Various MDM2 inhibitors, especially small-molecule inhibitors of the p53-MDM2 interaction, have shown anticancer activity. The most promising of these has been the small-molecule MDM2 antagonist nutlin-3a. This compound binds to the hydrophobic pocket in MDM2 protein normally occupied by the transactivation domain of p53, thus inhibiting the binding of p53 and activating the p53 pathway. Treatment with this small-molecule drug selectively induces cell cycle arrest, growth inhibition, and apoptosis in a variety of human cancers. However, the majority of these inhibitors exert their effects through restoring the p53 apoptotic response. Expression of wild-type p53 is specifically required. Cellular experiments with nutlin-3a confirmed that the response of tumor cells was tightly associated with the p53 status. Cell lines and primary cells harboring a p53 mutation or having a p53-null phenotype did not respond to these MDM2 antagonists [40]. But actually, as stated above, more than 50% of all solid tumors carry p53 mutation, and there is a higher incidence of p53 mutation or loss of p53 expression observed in aggressive cancer. Even with wild-type p53, activated p53 in turn induces expression of MDM2. Subsequently, this p53-mediated increase in MDM2 expression will begin to degrade p53 and repress its function, which is part of mechanism for the development of chemoresistance. In contrast, we now found that the activity of triptolide was not through p53. Triptolide could effectively inhibit MDM2 expression and showed potent anticancer and chemosensitization effects in different human breast cancer models with a different p53 status. Studies in breast cancer have indicated that mutations in p53 are predictive for worse prognosis and poor treatment outcome [39]. Thus, we believe that triptolide should be a promising new MDM2 inhibitor and particularly efficient for the treatment of high-risk, refractory breast cancer patients. In addition to triptolide, there have been several other naturally derived drugs that specifically target MDM2, such as berberine [41], curcumin [42] and parthenolide [43]. Future studies are necessary to explore the structure-activity relationship of these compounds, providing a new window into knowledge-based treatment for refractory cancers.

In summary, the results from the present study demonstrated that triptolide had anticancer and chemosensitization effects in human breast cancer by targeting MDM2/Akt. Our study helps to elucidate the p53-independent regulatory function of MDM2 in Akt signaling, offering a novel view of the mechanism by which triptolide functions as an anticancer agent.

## MATERIALS AND METHODS

### Cell culture

Two human breast cancer cell lines MCF-7 and MDA-MB-468 were used in this study. MCF-7 cell line has wild-type p53, while MDA-MB-468 cell line is p53-mutant. These two cell lines were obtained from China Center for Type Culture Collection (CCTCC). The cell lines were cultured in standard medium (RPMI 1640 medium containing 10% fetal bovine serum, 50 units/ml penicillin, and 50 μg/ml streptomycin) at 37°C under 5% CO_2_ in a humidified atmosphere.

### Reagents and cell treatment

Triptolide and doxorubicin were purchased from Sigma-Aldrich (St. Louis, MO, USA). Triptolide was dissolved in dimethyl sulfoxide (DMSO) to create a 10 mM stock solution and stored at −20°C. Doxorubicin was also prepared as a 10 mM stock solution. *In vitro* biological activity of triptolide was determined after incubation at various concentrations (25, 50, 75, 100 nM) for different periods of time as indicated (3, 6, 12, 24 h). Equivalent concentrations of DMSO were used as vehicle controls. To evaluate the *in vitro* chemosensitization effect of triptolide, MCF-7 and MDA-MB-468 cells were treated with doxorubicin alone or in combination with triptolide.

### Plasmids and gene transfection

To determine whether MDM2 plays a role in the reduction of Akt activation in triptolide-treated tumor cells, MDA-MB-468 cells were transfected with MDM2-specific small interfering RNA (siRNA) to silence the expression of MDM2, followed by exposure to triptolide. The pSUPER MDM2 siRNA plasmids were constructed by inserting several specific 19-nucleotide MDM2 sequence (clone1: ACACTTATACTATGAAAGA; clone2: GAAGTTATTAAAGTCTGTT; clone3: CAACATATTG TATATTGTT) into an expression plasmid, pSUPER-neo vector. For a control, a 19-nucleotide scrambled sequence (GAGGCTATTATACTGTGAT) was inserted into another pSUPER-neo vector. Silencing efficiency was detected by Western blotting. Gene transfection was also performed to investigate whether MDM2 inhibition plays a causative role in the *in vitro* anticancer activities and chemosensitization effect of triptolide. MDM2-expressing plasmid was introduced into tumor cells to induce overexpression of MDM2. After transfection, the cells were further treated with triptolide and then collected for effect analysis, including cell proliferation, cell apoptosis, and cell cycle distribution. Non-specific plasmid was co-transfected to provide an internal control. Transfection was done using Lipofectamine 2000 transfection kit from Invitrogen (CarIsbad, CA, USA) according to the manufacturer's instructions. For stable gene transfection, the cells were grown in medium containing G418 (300 μg/ml) for 2–3 weeks to select for the G418-resistant colonies that would be carrying the desired plasmids. The positive clones were confirmed by immunoblotting and maintained in medium containing G418.

### Xenograft model

This study was approved by the Animal Care and Use Committee of Huazhong University of Science and Technology. Experiment involving animals (BALB/c-nu/nu female mice, 4–6 weeks old) were obtained from the Experimental Animal Center of Tongji Medical College, Huazhong University of Science and Technology. The mice were maintained in a controlled environment (temperature 20–25°C, humidity 50–80%, illumination 12 h light/12 h dark). Mice were fed a standard laboratory food and water ad libitum for at least 1 week for environmental adjustment before study. MCF-7 or MDA-MB-468 cells were washed with serum-free medium and re-suspended in the same medium. The cell suspension (5 × 10^7^/ml, 0.1 ml) were injected subcutaneously into the left dorsal scapula region of the mice. The general conditions of the mice including body weight and physical status were observed daily. The mice were monitored by tumor growth. The tumor size was measured twice weekly for 6 weeks, and the volume of the tumor was calculated using the simplified formula: length × width^2^ × 0.5. The mice were sacrificed on day 42, and the tumors were resected and weighted. The inhibition rate of tumor growth on the basis of tumor weight was calculated according to the following formula: (1 – the average tumor weight of a treated group/that of control group) × 100%.

### *In vivo* treatment

Mice bearing MCF-7 or MDA-MB-468 xenografts were randomly divided into multiple treatment groups (10 mice/group). After 7 days, tumors were detected and each group of mice were given the following treatments: triptolide was administered intraperitoneally at a dose of 0.15 mg/kg/day for 14 days. Alone or in combination with triptolide, doxorubicin (2 mg/kg, once weekly, for 6 weeks) was administrated by intraperitoneal injection. The above doses and injection regimens were based on reports published previously [44–46].

### Quantitative RT-PCR

Quantitative RT-PCR was used to measure the expression of MDM2 at mRNA level. Expression of p85 transcript was also determined by quantitative RT-PCR analysis. Total RNA from cultured cells was isolated using Trizol reagent (Invitrogen) and reverse transcribed into cDNA with M-MLV (Invitrogen). Quantitative RT-PCR analysis was performed with SYBR Green qPCR kit (Roche Molecular Biochemicals, Mannheim, Germany) on Stratagene Mx3000 Quantitative RT-PCR System (Stratagene, La Jolla, CA, USA) according to the manufacturer's instructions. The primer sequences were as follows: MDM2 forward 5′-TGT TGGTGCACAAAAAGACACTT-3′, MDM2 reverse 5′-GCACGCCAAACAAATCTCCTA-3′; p85 forward 5′-TTGCGAGGGAAGCGAGATGGC-3′, p85 reverse 5′-TGCTGCACAAGGGAGGTGTGT-3′. The expression of target gene was normalized to that of GAPDH.

### Western blot analysis

The protein expression levels of MDM2, p53, p21, PUMA, Akt, phosphorylated Akt, Foxo3a, phosphorylated Foxo3a, PI3K, phosphorylated PI3K, p110, p85, REST, caspase-3, cleaved caspase-3 and PARP in the tumor cells were analyzed by Western blotting. Proteins were extracted in the lysis buffer [50 mM Tris-HCl (PH 7.2), 150 mM NaCl, 1% Triton X-100, 1% sodium deoxycholate, 0.1% SDS, 0.2 mM sodium vanadate, 1% phenylmethylsulfonyl fluoride, and 0.2% aprotinin]. Protein concentrations were measured by the BCA Protein Assay Kit (Pierce, Rockford, IL, USA). Equal amounts of protein extracts were resolved by SDS-PAGE and subsequently transferred to nitrocellulose membranes. After being blocked in 5% nonfat milk in TBST for 1 h, the membranes were incubated with primary mouse anti-MDM2 antibody (1:1000; Santa Cruz Biotechnology, Santa Cruz, CA, USA), primary mouse anti-p53 antibody (1:1000; Santa Cruz Biotechnology), primary rabbit anti-p21 antibody (1:1000; Cell Signaling Technology, Danvers, MA, USA), primary rabbit anti-PUMA antibody (1:1000; Santa Cruz Biotechnology), primary rabbit anti-Akt antibody (1:1000; Cell Signaling Technology), primary rabbit anti-phospho-Akt antibody (1:1000; Cell Signaling Technology), primary rabbit anti-Foxo3a antibody (1:1000; Cell Signaling Technology), primary rabbit anti-phospho-Foxo3a antibody (1:1000; Cell Signaling Technology), primary rabbit anti-PI3K antibody (1:1000; Cell Signaling Technology), primary rabbit anti-phospho-PI3K antibody (1:1000; Cell Signaling Technology), primary rabbit anti-p110 antibody (1:1000; Cell Signaling Technology), primary rabbit anti-p85 antibody (1:1000; Cell Signaling Technology), primary rabbit anti-REST antibody (1:1000; Millipore, Billerica, MA, USA), primary mouse anti-caspase-3 antibody (1:1000; Santa Cruz Biotechnology), primary rabbit anti-cleaved caspase-3 p11 antibody (1:1000; Santa Cruz Biotechnology), or primary rabbit anti-PARP antibody (1:1000; Cell Signaling Technology) overnight at 4°C. The membranes were then washed three times in TBST and incubated with horseradish peroxidase (HRP)-conjugated goat anti-rabbit or goat anti-mouse secondary antibody (1:3000). Finally, the signals were developed in enhanced chemiluminescence Kit (Millipore). GAPDH served as an internal control.

### Immunoprecipitation (IP)

Cells were lysed in a buffer composed of 50 mM Tris-HCl (pH 7.6), 150 mM NaCl, 1% NP-40, 10 mM sodium phosphate, 10 mM NaF, 1 mM sodium orthovanadate, 2 mM phenylmethylsulfonyl fluoride, 10 μg/ml aprotinin, 10 μg/ml leupeptin, and 10 μg/ml pepstatin. Cell lysates were immunoprecipitated with anti-MDM2 antibody (Santa Cruz) and isotype-matched control antibody plus protein A agarose overnight at 4°C. Whole cell lysates or immunoprecipitates were resolved by SDS-PAGE, transferred onto nitrocellulose membranes, and subsequently probed with anti-REST antibody (Millipore).

### Chromatin immunoprecipitation (CHIP) assay

Cells were incubated with 1% formaldehyde for 10 min at 37°C with mild shaking to crosslink protein-DNA complexes. Crosslinking was stopped by adding 200 mM glycine for 10 min at room temperature. Cells were then washed twice with cold phosphate-buffered saline and resuspended in lysis buffer (1% SDS, 10 mM EDTA, 50 mM Tris-HCl pH8.0) supplemented with protease inhibitors (1 mM phenylmethylsulfonyl fluoride, 1 μg/ml aprotinin and 1 μg/ml pepstatin). After incubated 10 min on ice, cells were sonicated to shear DNA to 200–1000 basepairs fragments. Sonicated lysates were cleared by centrifugation and were diluted 10-fold with dilution buffer (0.01% SDS, 1% Triton X-100, 1.2 mM EDTA, 16.7 mM Tris-HCl pH 8.0, 167 mM NaCl) containing protease inhibitors. Sonicated lysates were then immunoprecipitated overnight at 4°C using anti-REST antibody (Millipore) or control antibody. The resulting immunoprecipitated protein complexes were collected by protein A agarose saturated with bovine serum albumin and sheared salmon sperm DNA. Precipitants were sequentially washed once with a low salt wash buffer (0.1% SDS, 1% Triton X-100, 2 mM EDTA, 20 mM Tris-HCl pH 8.0, 150 mM NaCl), a high salt wash buffer (0.1% SDS, 1% Triton X-100, 2 mM EDTA, 20 mM Tris-HCl pH 8.0, 500 mM NaCl), and finally a LiCl wash buffer (0.25 M LiCl, 1% NP-40, 1% deoxycholate, 1 mM EDTA, 10 mM Tris-HCl pH 8.0), followed by two washes with 1 × TE buffer (20 mM Tris-HCl pH 8.0, 1 mM EDTA pH 8.0). Protein complexes were then eluted in elution buffer (1% SDS, 100 mM NaHCO_3_). 5 M NaCl was added to the combined eluates to reverse crosslinking by heating at 65°C overnight. Samples were ethanol precipitated and then resuspended in 1 × TE and incubated with 10 mg/ml RNase A for 30 min at 37°C and 10 mg/ml proteinase K for 1 h at 42°C. DNA was covered by phenol-chloroform extraction and ethanol precipitation. Finally, samples were resuspended in water and subjected to quantitative RT-PCR using a primer pair for amplification of the p85 promoter: forward 5′-GCGTCCGACCACACATGCCA-3′ and reverse 5′-GGGGGCAGAGGGGAGGAGTG-3′.

### Cell proliferation assay

The anti-proliferation effect of triptolide was determined by WST-1 assay. Briefly, cells were cultured in 96-well plates along with different concentrations of triptolide, alone or in combination with doxorubicin, for 24 h. WST-1 reagent (25 μg/well) (Roche) was then added into each well and the cells were continuously incubated for an additional 4 h. Following this, the optical density (OD) of the wells was read with a microplate reader (BioTek Instruments, Winooski, VT, USA) at a test wavelength of 450 nm and a reference wavelength of 620 nm. Appropriate controls lacking cells were included to determine background absorbance. All experiments were done in triplicate and repeated three times.

### Cell apoptosis assay

The ability of triptolide to induce apoptosis was determined by Annexin V staining and flow cytometry. After incubation with different concentrations of triptolide, cells were washed with PBS and then stained with FITC-Annexin V and propidium iodide (BD Pharmingen, San Diego, CA, USA) according to the manufacturer's instructions. Annexin V binds to the phosphatidylserine on the surface of apoptotic cells and propidium iodide stains the cellular DNA of those cells with compromised membrane. Apoptotic cells were detected on a FACScan flow cytometer using the CellQuest software (BD Biosciences, Mountain View, CA, USA).

### Cell cycle distribution analysis

Flow cytometry was also performed to analyze cell cycle distribution. Cells with or without triptolide treatment were fixed in 70% ethanol for 1 h at 4°C. The fixed cells were then washed with PBS and stained for DNA with 50 μg/ml of propidium iodide in the presence of 100 μg/ml of RNase for 30 min at 4°C. DNA contents of cells were finally determined by flow cytometry.

### Combination index analysis

MCF-7 and MDA-MB-468 cells were incubated with serial dilutions of triptolide and doxorubicin, either alone or in combination at a fixed ratio. Serial dilutions were made to span the individual IC_50_ of each drug. After 24 h of incubation, growth inhibition was measured by WST-1 assay. Median-effect plot analyses and calculation of the combination index (CI) were analyzed by the method of Chou and Talalay [47, 48]. Values statistically significantly less than 1.0 indicate synergistic interactions, values equal to 1.0 indicate additive interactions, and values greater than 1.0 indicate antagonistic interactions.

### Statistical analysis

At least three independent experiments were performed. All data were presented as mean ± SEM. Relative gene expression data were analyzed using the 2−ΔΔCT method [49]. Statistical significance for differences between groups was determined by one-way ANOVA or by the nonparametric Kruskal-Wallis test with the use of SPSS 13.0 software. All statistical tests were two-sided. The significance level was set at *P* < 0.05.
